# (μ-2,3-Dibromo­succinato-κ^2^ *O* ^1^:*O* ^4^)bis­[methano­lato-κ*O*)triphenyl­anti­mony(V)]

**DOI:** 10.1107/S1600536811016114

**Published:** 2011-05-07

**Authors:** Li Quan, Handong Yin, Wenfu Fu

**Affiliations:** a Technical Institute of Physics and Chemistry, Chinese Academy of Sciences, Beijing 100190, People’s Republic of China; b Graduate Unversity of the Chinese Academy of Sciences, Beijing 100049, People’s Republic of China; c College of Chemistry and Chemical Engineering, Liaocheng University, Shandong 252059, People’s Republic of China

## Abstract

In the title mol­ecule, [Sb_2_(C_6_H_5_)_6_(C_4_H_4_Br_2_O_4_)(CH_3_O)_2_], two [Sb(CH_3_O)Ph_3_]^+^ units are linked by the two carboxyl­ate O atoms of a *meso*-2,3-dibromo­succinate bridging ligand, forming a dinuclear compound. The Sb^IV^ atom is five-coordinated in a slightly distorted trigonal–bipyramid geometry by phenyl C atoms in the equatorial positions and two O atoms in the axial positions. C—H⋯O inter­actions link the mol­ecules into a two-dimensional network parallel to (010). The —CH— group of the centrosymmetric 2,3-dibromosuccinate anion is disordered over two sites in a 0.6:0.4 ratio.

## Related literature

For the synthesis and structural characteristics of related organo­phenyl­anti­mony compounds, see: Yin *et al.* (2008 ▶).
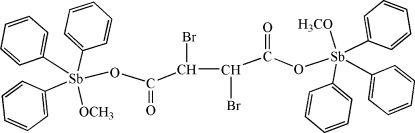


## Experimental

### 

#### Crystal data

[Sb_2_(C_6_H_5_)_6_(C_4_H_2_Br_2_O_4_)(CH_3_O)_2_]
*M* *_r_* = 1042.04
Triclinic, 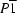

*a* = 8.707 (8) Å
*b* = 9.872 (8) Å
*c* = 12.779 (10) Å
α = 103.74 (2)°
β = 97.93 (2)°
γ = 100.45 (2)°
*V* = 1030.3 (15) Å^3^
*Z* = 1
Mo *K*α radiation
μ = 3.29 mm^−1^
*T* = 298 K
0.22 × 0.16 × 0.13 mm

#### Data collection

Siemens SMART CCD area-detector diffractometer
Absorption correction: multi-scan (*SADABS*; Sheldrick, 1996 ▶) *T*_min_ = 0.531, *T*_max_ = 0.674
5379 measured reflections
3582 independent reflections
2582 reflections with *I* > 2σ(*I*)
*R*_int_ = 0.032

#### Refinement

*R*[*F* ^2^ > 2σ(*F* ^2^)] = 0.048
*wR*(*F* ^2^) = 0.131
*S* = 1.00
3582 reflections
240 parameters
2 restraints
H-atom parameters constrained
Δρ_max_ = 1.10 e Å^−3^
Δρ_min_ = −0.68 e Å^−3^

### 

Data collection: *SMART* (Siemens, 1996 ▶); cell refinement: *SAINT* (Siemens, 1996 ▶); data reduction: *SAINT*; program(s) used to solve structure: *SHELXS97* (Sheldrick, 2008 ▶); program(s) used to refine structure: *SHELXL97* (Sheldrick, 2008 ▶); molecular graphics: *ORTEP-3* (Farrugia, 1997 ▶) and *DIAMOND* (Brandenburg, 1998 ▶); software used to prepare material for publication: *SHELXTL* (Sheldrick, 2008 ▶).

## Supplementary Material

Crystal structure: contains datablocks I, global. DOI: 10.1107/S1600536811016114/jh2282sup1.cif

Structure factors: contains datablocks I. DOI: 10.1107/S1600536811016114/jh2282Isup2.hkl

Additional supplementary materials:  crystallographic information; 3D view; checkCIF report

## Figures and Tables

**Table:** 

Sb1—O3	1.996 (5)
Sb1—C15	2.118 (7)
Sb1—C3	2.119 (7)
Sb1—C9	2.122 (7)
Sb1—O1	2.177 (4)

**Table:** 

O3—Sb1—C15	92.7 (2)
O3—Sb1—C3	87.8 (2)
O3—Sb1—C9	93.9 (3)
C15—Sb1—C9	119.3 (3)
C3—Sb1—C9	113.2 (3)
O3—Sb1—O1	176.33 (19)
C15—Sb1—O1	90.7 (2)
C3—Sb1—O1	91.2 (2)
C9—Sb1—O1	83.2 (2)

**Table 2:** Hydrogen-bond geometry (Å, °)

*D*—H⋯*A*	*D*—H	H⋯*A*	*D*⋯*A*	*D*—H⋯*A*
C19—H19⋯O2^i^	0.93	2.50	3.388 (11)	161
C17—H17⋯O3^ii^	0.93	2.59	3.435 (10)	152

Symmetry codes: (i) 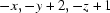; (ii) 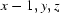.

**Fig. 1.**
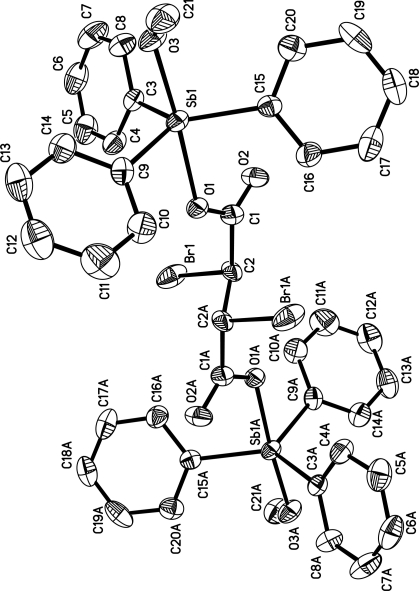
Molecular structure of compound (1) showing displacement ellipsoids drawn at the 30% probability level.

**Fig. 2.**
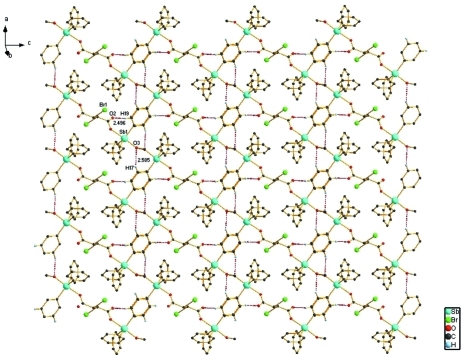
2D network structure infinitely by C19-H19···O2 and C17-H17···O3 interactions [Symmetry code: (i) -x,2-y,1-z; (ii) -1+x,+y,+z].

## supplementary crystallographic information

### Comment

The similar compound of organotin from meso-2,3-dibromosuccinic acid was synthesized by Professor Yin (Yin *et al.*, 2008). The Sb atom is five-coordinated (Figure 1) with an average Sb-C distance of 2.120 A° almost equal to the average Sn-C distance of 2.123 A° and average Sb-O distance of 2.087 A° much shorter than 2.182 A° of Sn-O distance (Yin *et al.*, 2008). The feature in the solid state structure is the 2D network structure infinitely linked by C19-H19···O2 (2.496 A° of H19···O2) and C17-H17···O3 (2.585 A° of H17···O3) interactions (Figure 2), while the organotin give a 3D network triorganitin(iv) polymer (Yin *et al.*, 2008). The title unit is sited in a symmetrical center, and the coordination environment around the antimony atom is described as a slightly distorted trigonal bipyramid with three carbon atoms from the discrete phenyls in equatorial positions and two oxygen atoms occupying the axial positions with O1-Sb1-O2 angle 176.33 (19)°. The SbC_3_ unit is planar (the sum of the C-Sb-C angle is 359.9 °) and the O-Sb-C angles are in the range of 83.2 (2)/93.9 (3)°.

### Experimental

2,3-dibromosuccinic acid (0.276 g, 1.00 mmol) was added to the solution of sodium methoxide (0.108 g, 2.00 mmol) in methanol (15 ml), and the mixture was refluxed with stirring for 30 min. Triphenylantimony chloride (0.423 g, 1.00 mmol) in toluene (25 ml) was then added to the mixture, and the reaction was allowed to continue for 8h under refluxing. After cooling down to room temperature, teh resulting solution was filtered. The solvent was removed by evaporation under vacuum leaving a coloeless solid. The product was recrystallized from dichloromethane/petroleum ether.

### Refinement

All H atoms were positioned geometrically and refined as riding atoms, with C—H = 0.93 A° , Uiso(H) = 1.2Ueq(C) and C—H = 0.96 A° / 0.98 A°, Uiso(H) = 1.5Ueq(C).

### Crystal data

**Table:**

[Sb_2_(C_6_H_5_)_6_(C_4_H_2_Br_2_O_4_)(CH_3_O)_2_]	*Z* = 1
*M**_r_* = 1042.04	*F*(000) = 510
Triclinic, *P*1	*D*_x_ = 1.679 Mg m^−^^3^
*a* = 8.707 (8) Å	Mo *K*α radiation, λ = 0.71073 Å
*b* = 9.872 (8) Å	Cell parameters from 1605 reflections
*c* = 12.779 (10) Å	θ = 2.4–25.2°
α = 103.74 (2)°	µ = 3.29 mm^−^^1^
β = 97.93 (2)°	*T* = 298 K
γ = 100.45 (2)°	Block, colorless
*V* = 1030.3 (15) Å^3^	0.22 × 0.16 × 0.13 mm

### Data collection

**Table:**

Siemens SMART CCD area-detector diffractometer	3582 independent reflections
Radiation source: fine-focus sealed tube	2582 reflections with *I* > 2σ(*I*)
graphite	*R*_int_ = 0.032
φ and ω scans	θ_max_ = 25.0°, θ_min_ = 1.7°
Absorption correction: multi-scan (*SADABS*; Sheldrick, 1996)	*h* = −10→10
*T*_min_ = 0.531, *T*_max_ = 0.674	*k* = −11→11
5379 measured reflections	*l* = −8→15

### Refinement

**Table:**

Refinement on *F*^2^	Primary atom site location: structure-invariant direct methods
Least-squares matrix: full	Secondary atom site location: difference Fourier map
*R*[*F*^2^ > 2σ(*F*^2^)] = 0.048	Hydrogen site location: inferred from neighbouring sites
*wR*(*F*^2^) = 0.131	H-atom parameters constrained
*S* = 1.00	*w* = 1/[σ^2^(*F*_o_^2^) + (0.0705*P*)^2^] where *P* = (*F*_o_^2^ + 2*F*_c_^2^)/3
3582 reflections	(Δ/σ)_max_ < 0.001
240 parameters	Δρ_max_ = 1.10 e Å^−^^3^
2 restraints	Δρ_min_ = −0.68 e Å^−^^3^

### Special details

**Table:**

Geometry. All esds (except the esd in the dihedral angle between two l.s. planes) are estimated using the full covariance matrix. The cell esds are taken into account individually in the estimation of esds in distances, angles and torsion angles; correlations between esds in cell parameters are only used when they are defined by crystal symmetry. An approximate (isotropic) treatment of cell esds is used for estimating esds involving l.s. planes.
Refinement. Refinement of F^2^ against ALL reflections. The weighted R-factor wR and goodness of fit S are based on F^2^, conventional R-factors R are based on F, with F set to zero for negative F^2^. The threshold expression of F^2^ > 2sigma(F^2^) is used only for calculating R-factors(gt) etc. and is not relevant to the choice of reflections for refinement. R-factors based on F^2^ are statistically about twice as large as those based on F, and R- factors based on ALL data will be even larger.

### Fractional atomic coordinates and isotropic or equivalent isotropic displacement parameters (Å^2^)

**Table:**

	*x*	*y*	*z*	*U*_iso_*/*U*_eq_	Occ. (<1)
Sb1	0.22561 (5)	0.81465 (5)	0.69951 (4)	0.04269 (19)	
Br1	0.22321 (10)	1.18121 (10)	1.08223 (7)	0.0758 (3)	
O1	0.1245 (5)	0.8897 (5)	0.8434 (4)	0.0449 (11)	
O2	0.0650 (6)	1.0844 (5)	0.8011 (4)	0.0593 (13)	
O3	0.3285 (6)	0.7419 (5)	0.5737 (4)	0.0595 (14)	
C1	0.0653 (9)	1.0029 (9)	0.8600 (6)	0.0528 (19)	
C2	0.028 (2)	1.0561 (18)	0.9733 (9)	0.045 (3)	0.60 (2)
H2	−0.0534	1.1125	0.9678	0.054*	0.60 (2)
C2'	−0.043 (2)	1.001 (3)	0.9429 (16)	0.045 (3)	0.40 (2)
H2'	−0.0900	1.0854	0.9512	0.054*	0.40 (2)
C3	0.4220 (8)	0.9923 (7)	0.7547 (6)	0.0433 (16)	
C4	0.4634 (8)	1.0663 (8)	0.8653 (7)	0.059 (2)	
H4	0.4005	1.0413	0.9140	0.071*	
C5	0.5985 (10)	1.1778 (9)	0.9040 (8)	0.074 (3)	
H5	0.6257	1.2256	0.9783	0.089*	
C6	0.6886 (10)	1.2161 (10)	0.8353 (9)	0.077 (3)	
H6	0.7770	1.2918	0.8612	0.092*	
C7	0.6504 (11)	1.1430 (11)	0.7251 (10)	0.086 (3)	
H7	0.7145	1.1688	0.6774	0.103*	
C8	0.5161 (9)	1.0307 (8)	0.6851 (7)	0.062 (2)	
H8	0.4911	0.9822	0.6109	0.074*	
C9	0.2537 (8)	0.6454 (7)	0.7711 (6)	0.0472 (17)	
C10	0.1370 (10)	0.5804 (8)	0.8189 (7)	0.069 (2)	
H10	0.0415	0.6098	0.8200	0.083*	
C11	0.1654 (14)	0.4720 (10)	0.8645 (9)	0.096 (3)	
H11	0.0868	0.4269	0.8950	0.115*	
C12	0.3079 (14)	0.4287 (10)	0.8658 (9)	0.086 (3)	
H12	0.3252	0.3558	0.8975	0.103*	
C13	0.4230 (12)	0.4940 (10)	0.8202 (8)	0.079 (3)	
H13	0.5202	0.4673	0.8225	0.094*	
C14	0.3953 (9)	0.6001 (8)	0.7704 (7)	0.061 (2)	
H14	0.4721	0.6410	0.7364	0.074*	
C15	0.0083 (8)	0.7988 (7)	0.5950 (5)	0.0427 (16)	
C16	−0.1375 (9)	0.7702 (8)	0.6290 (7)	0.059 (2)	
H16	−0.1398	0.7584	0.6989	0.071*	
C17	−0.2776 (10)	0.7594 (10)	0.5594 (9)	0.077 (3)	
H17	−0.3734	0.7400	0.5832	0.092*	
C18	−0.2792 (12)	0.7763 (10)	0.4578 (9)	0.076 (3)	
H18	−0.3747	0.7696	0.4121	0.091*	
C19	−0.1357 (13)	0.8041 (9)	0.4223 (7)	0.079 (3)	
H19	−0.1355	0.8148	0.3520	0.095*	
C20	0.0100 (10)	0.8161 (8)	0.4921 (7)	0.060 (2)	
H20	0.1057	0.8356	0.4682	0.072*	
C21	0.2653 (11)	0.6019 (9)	0.5003 (7)	0.076 (3)	
H21A	0.1873	0.5493	0.5306	0.114*	
H21B	0.3498	0.5524	0.4905	0.114*	
H21C	0.2167	0.6103	0.4307	0.114*	

### Atomic displacement parameters (Å^2^)

**Table:**

	*U*^11^	*U*^22^	*U*^33^	*U*^12^	*U*^13^	*U*^23^
Sb1	0.0395 (3)	0.0443 (3)	0.0438 (3)	0.0105 (2)	0.0141 (2)	0.0065 (2)
Br1	0.0568 (5)	0.0780 (6)	0.0655 (6)	−0.0172 (4)	0.0232 (4)	−0.0143 (5)
O1	0.048 (3)	0.049 (3)	0.043 (3)	0.021 (2)	0.016 (2)	0.013 (2)
O2	0.080 (4)	0.061 (3)	0.048 (3)	0.023 (3)	0.027 (3)	0.022 (3)
O3	0.050 (3)	0.061 (3)	0.062 (3)	0.012 (2)	0.023 (3)	−0.002 (3)
C1	0.055 (4)	0.066 (5)	0.044 (4)	0.020 (4)	0.018 (4)	0.017 (4)
C2	0.057 (10)	0.055 (11)	0.025 (6)	0.025 (7)	0.006 (7)	0.004 (6)
C2'	0.057 (10)	0.055 (11)	0.025 (6)	0.025 (7)	0.006 (7)	0.004 (6)
C3	0.038 (4)	0.044 (4)	0.049 (4)	0.012 (3)	0.011 (3)	0.011 (3)
C4	0.044 (4)	0.067 (5)	0.060 (5)	0.008 (4)	0.013 (4)	0.006 (4)
C5	0.057 (5)	0.070 (6)	0.077 (6)	0.007 (4)	−0.005 (5)	0.000 (5)
C6	0.045 (5)	0.061 (6)	0.109 (8)	0.002 (4)	0.004 (5)	0.008 (6)
C7	0.063 (6)	0.082 (7)	0.121 (9)	0.006 (5)	0.046 (6)	0.034 (7)
C8	0.057 (5)	0.060 (5)	0.070 (6)	0.009 (4)	0.027 (4)	0.018 (4)
C9	0.044 (4)	0.038 (4)	0.051 (4)	0.007 (3)	−0.001 (4)	0.004 (3)
C10	0.065 (5)	0.054 (5)	0.091 (7)	0.010 (4)	0.014 (5)	0.030 (5)
C11	0.105 (8)	0.070 (7)	0.108 (9)	−0.005 (6)	0.014 (7)	0.038 (6)
C12	0.100 (8)	0.052 (6)	0.100 (8)	0.015 (6)	−0.006 (7)	0.023 (5)
C13	0.082 (6)	0.062 (6)	0.088 (7)	0.028 (5)	−0.007 (6)	0.017 (5)
C14	0.061 (5)	0.052 (5)	0.069 (6)	0.017 (4)	0.013 (4)	0.010 (4)
C15	0.048 (4)	0.038 (4)	0.037 (4)	0.006 (3)	0.010 (3)	0.002 (3)
C16	0.054 (5)	0.071 (5)	0.051 (5)	0.019 (4)	0.015 (4)	0.008 (4)
C17	0.044 (5)	0.078 (6)	0.096 (8)	0.015 (4)	0.005 (5)	0.001 (6)
C18	0.069 (6)	0.069 (6)	0.080 (7)	0.018 (5)	−0.018 (5)	0.020 (5)
C19	0.116 (8)	0.067 (6)	0.043 (5)	0.015 (6)	−0.012 (6)	0.014 (4)
C20	0.063 (5)	0.054 (5)	0.057 (5)	0.000 (4)	0.010 (4)	0.014 (4)
C21	0.081 (6)	0.072 (6)	0.068 (6)	0.023 (5)	0.025 (5)	−0.003 (5)

### Geometric parameters (Å, °)

**Table:**

Sb1—O3	1.996 (5)	C8—H8	0.9300
Sb1—C15	2.118 (7)	C9—C14	1.386 (10)
Sb1—C3	2.119 (7)	C9—C10	1.391 (10)
Sb1—C9	2.122 (7)	C10—C11	1.378 (12)
Sb1—O1	2.177 (4)	C10—H10	0.9300
Br1—C2	2.032 (18)	C11—C12	1.383 (14)
Br1—C2'^i^	2.09 (2)	C11—H11	0.9300
O1—C1	1.299 (8)	C12—C13	1.366 (14)
O2—C1	1.225 (8)	C12—H12	0.9300
O3—C21	1.435 (9)	C13—C14	1.387 (11)
C1—C2'	1.515 (10)	C13—H13	0.9300
C1—C2	1.521 (9)	C14—H14	0.9300
C2—C2^i^	1.48 (3)	C15—C20	1.367 (10)
C2—H2	0.9800	C15—C16	1.401 (10)
C2'—C2'^i^	1.56 (5)	C16—C17	1.378 (11)
C2'—Br1^i^	2.09 (2)	C16—H16	0.9300
C2'—H2'	0.9800	C17—C18	1.346 (13)
C3—C8	1.362 (10)	C17—H17	0.9300
C3—C4	1.392 (10)	C18—C19	1.390 (13)
C4—C5	1.397 (11)	C18—H18	0.9300
C4—H4	0.9300	C19—C20	1.414 (12)
C5—C6	1.330 (13)	C19—H19	0.9300
C5—H5	0.9300	C20—H20	0.9300
C6—C7	1.383 (14)	C21—H21A	0.9600
C6—H6	0.9300	C21—H21B	0.9600
C7—C8	1.397 (12)	C21—H21C	0.9600
C7—H7	0.9300		
			
O3—Sb1—C15	92.7 (2)	C3—C8—C7	120.0 (8)
O3—Sb1—C3	87.8 (2)	C3—C8—H8	120.0
C15—Sb1—C3	127.3 (3)	C7—C8—H8	120.0
O3—Sb1—C9	93.9 (3)	C14—C9—C10	119.6 (7)
C15—Sb1—C9	119.3 (3)	C14—C9—Sb1	117.8 (6)
C3—Sb1—C9	113.2 (3)	C10—C9—Sb1	122.6 (5)
O3—Sb1—O1	176.33 (19)	C11—C10—C9	118.9 (8)
C15—Sb1—O1	90.7 (2)	C11—C10—H10	120.5
C3—Sb1—O1	91.2 (2)	C9—C10—H10	120.5
C9—Sb1—O1	83.2 (2)	C10—C11—C12	121.6 (10)
C2—Br1—C2'^i^	37.5 (5)	C10—C11—H11	119.2
C1—O1—Sb1	123.3 (4)	C12—C11—H11	119.2
C21—O3—Sb1	120.8 (5)	C13—C12—C11	119.3 (9)
O2—C1—O1	125.4 (6)	C13—C12—H12	120.3
O2—C1—C2'	121.6 (11)	C11—C12—H12	120.3
O1—C1—C2'	111.1 (11)	C12—C13—C14	120.2 (9)
O2—C1—C2	116.6 (8)	C12—C13—H13	119.9
O1—C1—C2	116.5 (8)	C14—C13—H13	119.9
C2'—C1—C2	28.0 (7)	C9—C14—C13	120.4 (8)
C2^i^—C2—C1	115.5 (14)	C9—C14—H14	119.8
C2^i^—C2—Br1	103.3 (13)	C13—C14—H14	119.8
C1—C2—Br1	111.7 (10)	C20—C15—C16	119.3 (7)
C2^i^—C2—H2	108.7	C20—C15—Sb1	119.5 (5)
C1—C2—H2	108.7	C16—C15—Sb1	121.2 (5)
Br1—C2—H2	108.7	C17—C16—C15	120.3 (8)
C1—C2'—C2'^i^	113.1 (18)	C17—C16—H16	119.9
C1—C2'—Br1^i^	118.2 (14)	C15—C16—H16	119.9
C2'^i^—C2'—Br1^i^	97.0 (18)	C18—C17—C16	121.7 (9)
C1—C2'—H2'	109.3	C18—C17—H17	119.2
C2'^i^—C2'—H2'	109.3	C16—C17—H17	119.2
Br1^i^—C2'—H2'	109.3	C17—C18—C19	118.9 (8)
C8—C3—C4	118.6 (7)	C17—C18—H18	120.5
C8—C3—Sb1	121.3 (6)	C19—C18—H18	120.5
C4—C3—Sb1	120.0 (5)	C18—C19—C20	120.7 (8)
C3—C4—C5	120.6 (8)	C18—C19—H19	119.7
C3—C4—H4	119.7	C20—C19—H19	119.7
C5—C4—H4	119.7	C15—C20—C19	119.2 (8)
C6—C5—C4	120.5 (9)	C15—C20—H20	120.4
C6—C5—H5	119.8	C19—C20—H20	120.4
C4—C5—H5	119.8	O3—C21—H21A	109.5
C5—C6—C7	119.9 (9)	O3—C21—H21B	109.5
C5—C6—H6	120.1	H21A—C21—H21B	109.5
C7—C6—H6	120.1	O3—C21—H21C	109.5
C6—C7—C8	120.4 (9)	H21A—C21—H21C	109.5
C6—C7—H7	119.8	H21B—C21—H21C	109.5
C8—C7—H7	119.8		

Symmetry codes: (i) −*x*, −*y*+2, −*z*+2.

### Hydrogen-bond geometry (Å, °)

**Table:**

*D*—H···*A*	*D*—H	H···*A*	*D*···*A*	*D*—H···*A*
C19—H19···O2^ii^	0.93	2.50	3.388 (11)	161
C17—H17···O3^iii^	0.93	2.59	3.435 (10)	152

Symmetry codes: (ii) −*x*, −*y*+2, −*z*+1; (iii) *x*−1, *y*, *z*.
